# Effect of Quenching Temperature on Microstructure and Hydrogen-Induced Cracking Susceptibility in S355 Steel

**DOI:** 10.3390/ma18051161

**Published:** 2025-03-05

**Authors:** Chunyan Yan, Shenglin Zhang, Lingchuan Zhou, Zhanpeng Tian, Mengdie Shen, Xinyi Liu

**Affiliations:** College of Materials Science and Engineering, Hohai University, Changzhou 213022, China; 221319020005@hhu.edu.cn (S.Z.); 231325020007@hhu.edu.cn (L.Z.); 2161410111@hhu.edu.cn (Z.T.); 2161410203@hhu.edu.cn (M.S.); 2261410203@hhu.edu.cn (X.L.)

**Keywords:** S355 steel, heat treatment, hydrogen-induced cracking, hydrogen permeation, hydrogen microprint technique

## Abstract

S355 steels are widely used in various applications. However, they may be affected by hydrogen, which can induce hydrogen-induced cracking (HIC). The effects of the quenching temperature (*T*_wq_) on the microstructure variation and HIC susceptibility of S355 steel was investigated by microstructural characterization, hydrogen permeation (HP) test, slow strain rate tensile (SSRT) test, hydrogen microprint technique (HMT) test, and hydrogen-charged cracking test. The results indicate that the microstructure of the treated specimens consisted of predominantly lath martensite (LM) and small amounts of lath bainite (LB) for the *T*_wq_ of 950 °C and 1000 °C, while the microstructure of the treated specimens mainly consisted of LM for the *T*_wq_ of 1050 °C and 1100 °C. The results indicate that as the *T*_wq_ increased, the sample treated at 950 °C exhibited a minimum hydrogen embrittlement index (*I*_z_), while the sample treated at 1050 °C exhibited the maximum *I*_z_. The hydrogen diffusion coefficient was relatively low, while the hydrogen concentration and trap density were relatively high for the *T*_wq_ of 1050 °C. The lath interfaces in martensite were effective hydrogen traps with high hydrogen-trapping efficiency. Hydrogen-induced cracks were significantly affected by hydrogen trapping at martensitic lath interfaces, exhibiting a basically transgranular fracture.

## 1. Introduction

Hydrogen is regarded as an ideal sustainable energy source for the future due to its excellent properties, including high energy density, clean emissions, and abundant availability. However, due to their small size and high reactivity, hydrogen atoms can diffuse through channels such as lattice interstices or lattice defects in the steel lattice. In practical engineering, hydrogen may infiltrate industrial equipment at various stages of production or operation process, which causes the premature failure of steel structures [1,2]. The HIC phenomenon, first reported in the 19th century, was induced by the infiltration of hydrogen atoms into metallic materials [3,4]. Hydrogen atoms typically originate from the environment in which the material is located, and they can also be introduced during the corrosion process or during the manufacturing or processing of the materials (such as welding, pickling, etc.) [5]. Once hydrogen atoms enter the metal, they may diffuse and accumulate within the microstructure of the metal, which is the HIC phenomenon. The presence of hydrogen will alter the internal stress state of metals, inducing the weakening of the interatomic bonding forces. Hydrogen tends to accumulate in specific areas, such as inclusions, grain boundaries, and dislocations, resulting in regions of high concentration [6]. In these regions, hydrogen can lead to an increase in localized plastic deformation, triggering the initiation of microcracks [3,7]. With the continuous accumulation and action of hydrogen, the microcracks gradually propagate and connect, eventually leading to macroscopic cracking. The HIC susceptibility in steel is affected by various factors like mechanical properties, microstructures, crystallographic characteristics, and inclusions [8,9,10]. These factors influence the amount, distribution, and mobility of hydrogen atoms within the steel [11].

Heat treatment is a commonly used metalworking process to study the variation in microstructures, mechanical properties, corrosion resistance, and HIC properties of low-alloy high-strength (HSLA) steels [12]. Yanachkov [13] investigated the effect of hydrogen concentration on the tensile properties of quenched S355 steel and found that as the hydrogen concentration increased, the fracture mode transitioned from ductile to brittle. Zhang [14] investigated the HIC susceptibility of Q500 steel and discovered that the dislocation and grain boundary density were greatly reduced in the normalized steel plate. Gao [15] investigated the effect of grain size on the HIC susceptibility in high-strength steels. The results showed that grain refinement increased the number of hydrogen traps and reduced hydrogen segregation in the steel, which resulted in a decrease in the HIC susceptibility of the steel. Anijdan [5] investigated the relationship between the microstructural variations and the HIC susceptibility in X65 pipeline steel at different *T*_wq_ levels. The research found that at lower *T*_wq_ levels, the HIC susceptibility in steel increased significantly with rising pearlite content and decreasing ferrite content.

Existing research has indeed achieved certain results in the study of heat-treated HSLA steels. However, most of these studies are limited to exploring individual factors in isolation. Under various *T*_wq_ levels, these factors do not act independently in quenched steels; instead, they interact in a complex manner, and this aspect has not been systematically investigated. The aim of this study was to acquire a clear interpretation of the effect of grain size and microstructure variation on hydrogen diffusion and HIC susceptibility in quenched steels. S355 steel is a typical HSLA steel. In terms of its mechanical properties, it has a minimum yield strength from 355 MPa, with a tensile strength ranging from 470~630 MPa and an elongation after fracture of 20~25%. These outstanding mechanical characteristics contribute to its excellent performance. Additionally, it also exhibits a good welding performance [16,17] and it is widely used in the manufacturing of buildings, bridges, pressure vessels, and marine structures [18,19,20]. In the oil and gas industry, S355 steel is commonly used as structural steel. During the extraction and transportation of oil and gas, hydrogen-containing substances may cause hydrogen atoms to enter the steel matrix through electrochemical reactions. Additionally, in the hydrogen energy field, for some low-pressure hydrogen storage facilities and their supporting structures made of S355 steel, hydrogen may also diffuse into the steel. Owing to the extensive application of S355 steel in key engineering fields and given its relatively simple alloy composition and well-defined basic properties, it serves as an ideal candidate for investigating the effects of water-quenching at varying temperatures on its microstructure and HIC susceptibility. In this study, S355 steel was water-quenched at different temperatures to obtain a basically martensitic microstructure with different grain sizes. HIC susceptibility and hydrogen trapping in S355 steels at different *T*_wq_ levels were investigated using microstructural observation, SSRT test, HP test, and HMT test. The results will provide a theoretical basis for the application of corrosion protection in HSLA steels.

## 2. Materials and Methods

### 2.1. Experimental Materials

**Figure 1:**
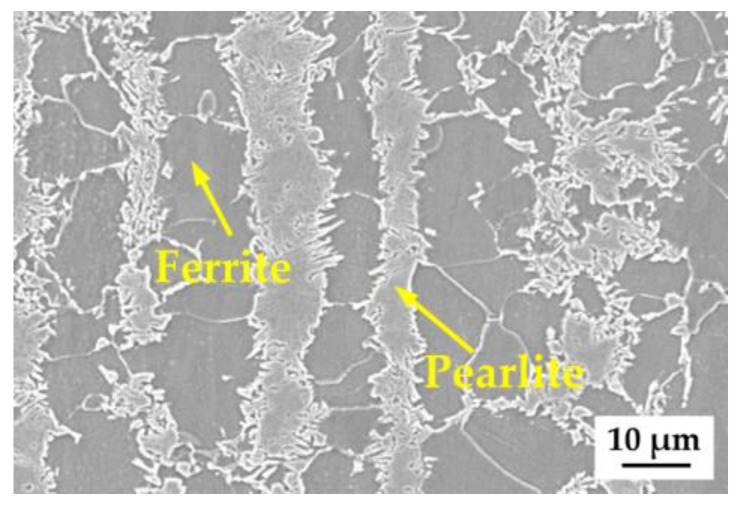
Scanning electron microscopy image of microstructure in S355 steel as-received.

In this study, samples for heat treatment were cut from the middle section of hot-rolled HSLA S355 steel produced by a domestic steel company (BAOSTEEL, Shanghai, China). The chemical composition and mechanical properties are shown in Table 1 and Table 2, respectively. After being etched with a 4% nital solution for 5~10 s, the samples revealed a microstructure consisting of banded pearlite (P) and ferrite (F), which is presented in Figure 1.

**Figure 2:**
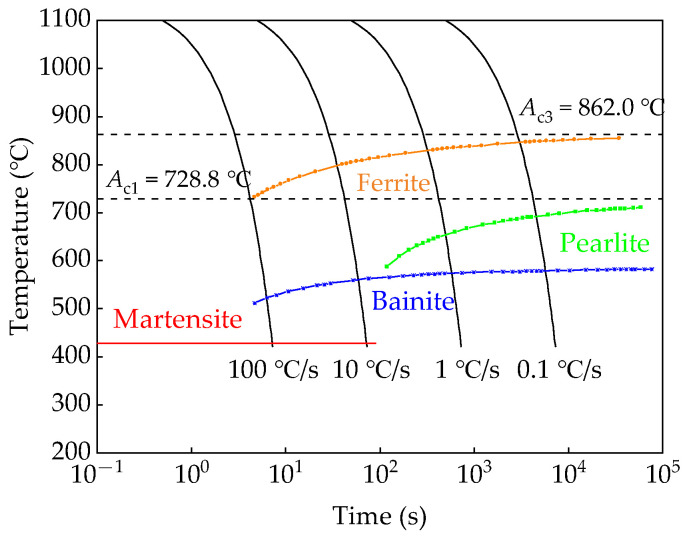
Simulated CCT diagram for the tested S355 steel using the JmatPro software program.

**Figure 3:**
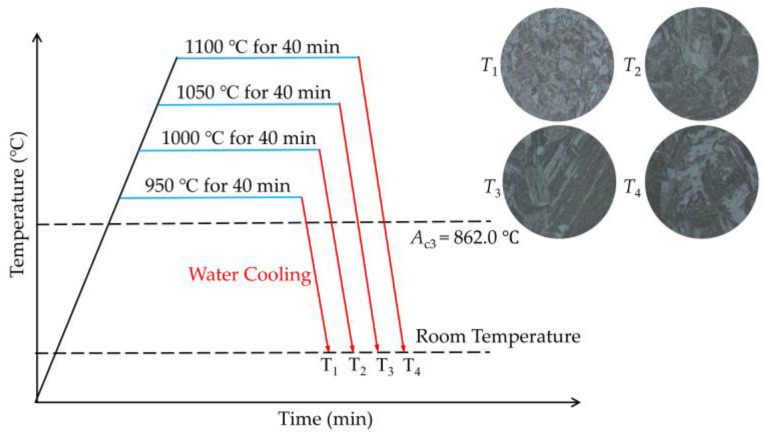
Heat treatment process applied to S355 Steel.

Before heat treatment, a thermodynamic analysis of the material was conducted. Figure 2 shows the CCT diagram of the tested S355 calculated by the JMatPro 7.0 software program. As shown in Figure 2, the start and end temperatures of austenite transformation (*A*_c1_ and *A*_c3_) for S355 steel are 728.8 °C and 862.0 °C, respectively. Typically, full austenitization can be achieved above the *A*_c3_. When the austenitization temperature exceeds 1200 °C, the austenite grains will significantly coarsen, potentially leading to overheating, which negatively impacts the material’s performance. The austenitization temperatures for the test steels were set below 1200 °C to study the microstructure variation and its effect on the hydrogen diffusion behavior and HIC sensitivity of the heat-treated materials. The heat treatment process is shown in Figure 3. Samples measuring 60 mm × 20 mm × 15 mm were cut from the S355 steel plate. These samples were austenitizated in the heat treatment furnace at four temperatures for 40 min, which were designated as *T*_1_ (950 °C), *T*_2_ (1000 °C), *T*_3_ (1050 °C), and *T*_4_ (1100 °C), respectively. Then, the four samples were quenched in water with the ambient temperature of 20 °C to achieve hard microstructures consisting mainly of martensite.

### 2.2. Microstructure Observation and Hardness Test

Metallographic samples measuring 17 mm × 13 mm × 10 mm were cut from the heat-treated steels using wire cutting. The samples were ground using 240~2000 grit SiC papers and polished with a diamond paste (W1.5) on woolen fabric. Then, they were etched with 4% nital solution for 5~10 s. After etching, the samples were rinsed with anhydrous ethanol and dried. Microstructure observations were conducted using a Zeiss Axiocam 105 color optical microscope (Carl Zeiss, Oberkochen, Germany) and a Quanta 250FEG (FEI, New York, NY, USA) scanning electron microscope (SEM). A grain size analysis was performed on the metallographic samples. The samples were etched for 3~5 min at 70 °C with a grain size etchant. After etching, the grain size observation was conducted using a Zeiss Axiocam 105 color optical microscope (Carl Zeiss, Oberkochen, Germany) to obtain at least 20 images for each sample. The average grain sizes for the samples were measured based on the optical images using Image Pro Plus 6.0 software. Hardness tests were conducted using a Huayin HV-1000 microhardness tester (Laizhou Huayin, Laizhou, China). For each sample, 10 measurements were taken at different surface locations with a 1.96 N load applied for 20 s.

### 2.3. Pre-Charged Hydrogen Slow Strain Rate Tensile Test

**Figure 4:**
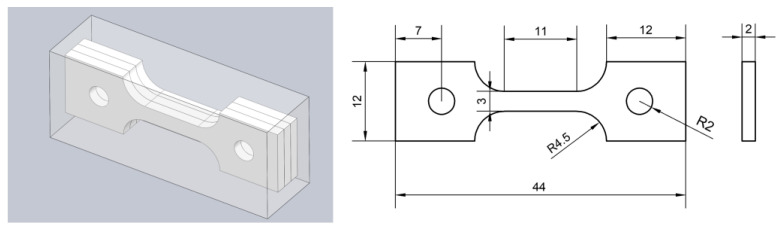
Tensile sample dimension diagram (in mm).

The SSRT tests were carried out following the ASTM E8 standard. For each group of samples, 3 specimens were tested. The samples were cut from the heat-treated steels using wire cutting, and the geometry of the samples is shown in Figure 4. They were pre-charged with hydrogen using CS1002 potentiostat/galvanostat (Wuhan Corrtest, Wuhan, China). The H-charging was conducted at room temperature, which was approximately 20 °C. The solution was a mixture of 0.5 mol/L H_2_SO_4_ and 0.5 g/L CH_4_N_2_S. The current density was 50 mA/cm^2^ and the time was 4 h. Based on previous experience and results of preliminary tentative experiments on hydrogen charging duration, the hydrogen charging time was determined to be 4 h. After H-charging, the samples were rinsed with anhydrous ethanol and dried. Then, they were shifted to a Letry WDML-50 microcomputer-controlled machine (Letry, Xi’an, China). The SSRT tests were conducted immediately with a constant strain rate of 1.1 × 10^−7^ m/s. The specimen elongation was monitored using an extensometer. Fracture morphology observation was carried out using a JSM-6510 (JEOL Ltd., Tokyo, Japan) SEM. The hydrogen embrittlement index *I*_z_ of the samples can be calculated according to the formula in the literature [21].

(1) $$I_{Z}=\frac{Z_{0}-Z_{B}}{Z_{0}}$$

In Equation (1), *I*_z_ is the HE index, *Z*_0_ is the section shrinkage of the uncharged sample, and *Z*_B_ is the section shrinkage of the charged sample.

The tensile strength loss *R*_mloss_ can be calculated according to the following formula:

(2) $$R_{mloss}=\frac{T_{0}-T}{T_{0}}$$

In Equation (2), *T*_0_ represents the initial tensile strength of the uncharged sample, and *T* represents the tensile strength of the charged sample.

The elongation loss *A*_loss_ can be calculated according to the formula:

(3) $$A_{loss}=\frac{E_{0}-E}{E_{0}}$$

In Equation (3), *E*_0_ represents the initial elongation of the uncharged sample, and *E* represents the elongation of the charged sample.

### 2.4. Hydrogen Permeation Test

**Figure 5:**
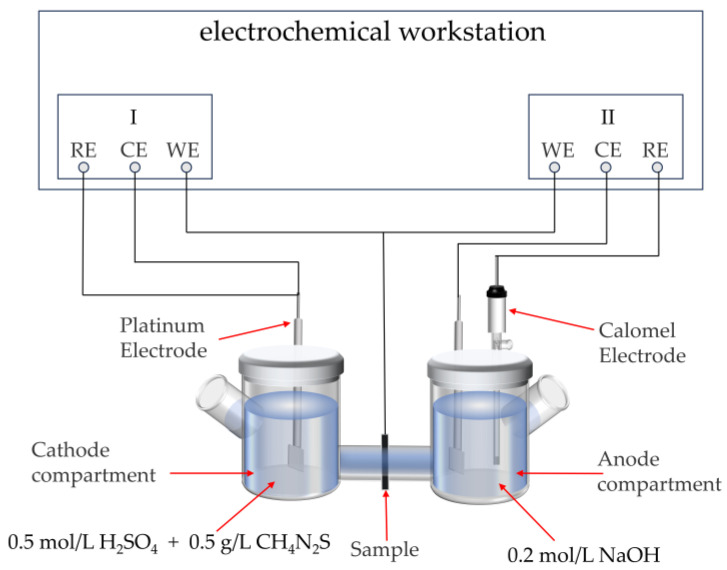
Diagram of hydrogen permeation test device.

Two-cycle HP tests for each group of water-quenched samples were carried out according to the ASTM G148 standard. In each group of hydrogen permeation tests, three samples were prepared, and each sample was subjected to the hydrogen permeation test twice. Samples measuring 18 mm × 12 mm × 1 mm were cut from heat-treated steels using wire cutting. The samples were ground and polished on both sides using SiC papers and diamond polishing paste. Nickel plating on one side was performed using a CS2350 electrochemical workstation (Wuhan Corrtest, Wuhan, China) with a current density of 4 mA/cm^2^ for 360 s to achieve a nickel coating thickness close to 0.2 μm. The permeation tests were carried out at room temperature, which was approximately 20 °C. As shown in Figure 5, the HP tests were conducted employing a CS2350 electrochemical workstation and a Devanathan–Stachurski double electrochemical cell setup [22]. The anode compartment contains a 0.2 mol/L NaOH solution. When hydrogen atoms reach the anode surface, they are oxidized to H^+^, which immediately combine with OH⁻ to form water (H^+^ + OH^−^ → H_2_O). An anodic potential of +300 mV was applied to ensure the complete ionization of diffusible atomic hydrogen in the samples. The test results were considered unaffected by residual diffusible hydrogen when the background current stabilized below 1 × 10⁻⁶ A. After the background current was eliminated, the hydrogen was charged, and the real-time hydrogen charging current density–time curve of the anode was observed. When the power supply was turned off after reaching the steady state for a period of time, the anode current began to decay. When it dropped to 2 × 10^−7^ A, the first cycle test was completed. Then, the above operation and completed the test of the second cycle was repeated. After completing the tests, four parameters can be calculated based on Fick’s law: HP flux (*J*_∞_), hydrogen effective diffusion coefficient (*D*_eff_), hydrogen concentration (*C*_0_), and hydrogen trap density (*N*_T_) [23,24].

In the first cycle, the hydrogen traps in the sample included both irreversible and reversible traps. Therefore, the trap density calculated from the first test represents the total hydrogen trap density. The second cycle only reflects the effect of reversible hydrogen traps, and the calculated value is the density of reversible hydrogen traps (*N*_r_). Based on the obtained *N*_T_ and *N*_r_, the irreversible hydrogen trap density (*N*_ir_) was calculated.

### 2.5. Hydrogen Microprint Technique Test

The HMT test is a method invented by Ovejero-García for observing the diffusion distribution of hydrogen in metals [25]. The test replaces hydrogen in the metal by utilizing the reaction between hydrogen and AgBr (AgBr + H → Ag + HBr). This method enables the hydrogen escape site to be visualized as silver particles. HMT samples of 18 mm × 12 mm × 1 mm were cut from the heat-treated steels. The samples were polished and ground on both sides and hydrogen was charged on one side using a CS1002 galvanostat. The charging solution was 0.5 mol/L H_2_SO_4_ with 0.5 g/L CH_4_N_2_S, and the current density was 50 mA/cm^2^ for 15 min. Then, the uncharged side of sample was etched, followed by cleaning with anhydrous ethanol. The etched side of the sample was placed facing down in a glass dish containing 1 mol/L AgBr solution and 1 mol/L NaNO_2_ solution in a dark environment. Since AgBr is prone to decomposition upon exposure to light (2AgBr → 2Ag + Br_2_), it is necessary to conduct the experiment in a dark environment to avoid interference from the decomposition of AgBr on the experimental results. The glass dish was placed in an HH-2 water bath (Lichen, Shanghai, China) and heated for 15 min at 70 °C. A microstructure observation and an EDS analysis of the samples’ corroded surface were conducted using a Quanta 250FEG SEM (FEI, New York, NY, USA).

### 2.6. Hydrogen-Charged Cracking Test

**Figure 6:**
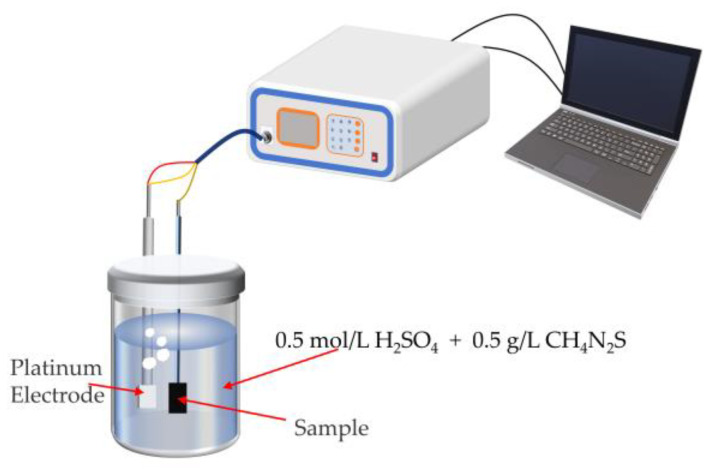
Schematic of the hydrogen-charged cracking setup.

Metal slices of 18 mm × 13 mm × 2 mm were cut from the water-quenched samples and polished on both sides. The hydrogen was charged using a CS1002 galvanostat. The test setup is shown in Figure 6. The auxiliary electrode is connected to a platinum electrode, while the working electrode is connected to the sample. Electrochemical hydrogen charging was conducted with a current density of 100 mA·cm^2^ for 3 h to induce cracking. The hydrogen charging time was determined based on previous experience and results of preliminary tentative experiments. In each group of the hydrogen-charged cracking test, three specimens were selected for measurement. After hydrogen charging was completed, the samples were then rinsed with anhydrous ethanol and etched using a 4% nital solution for 5 to 10 s. Crack propagation observation of the hydrogen-charged surface was conducted using a Quanta 250FEG SEM.

## 3. Results

### 3.1. Microstructure and Hardness

**Figure 7:**
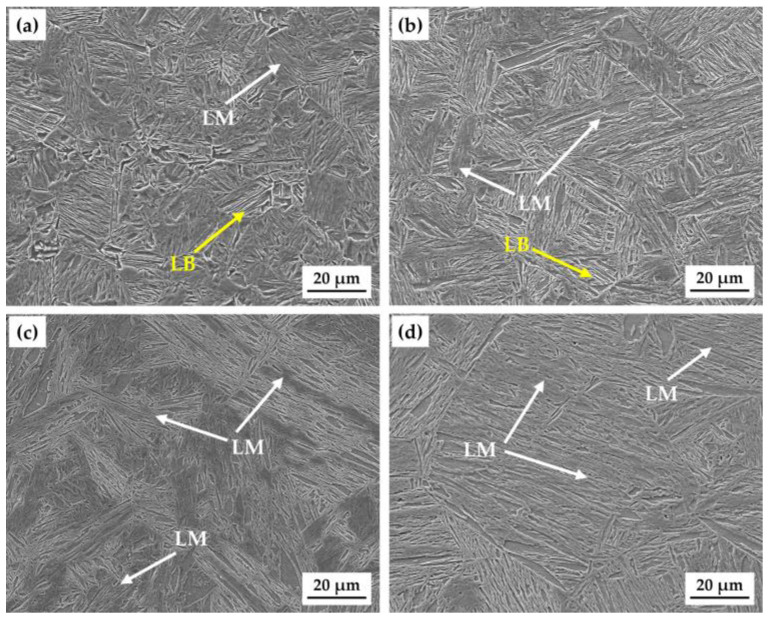
Microstructures of the test steels at different *T*_wq_ levels: (**a**) 950 °C, (**b**) 1000 °C, (**c**) 1050 °C, (**d**) 1100 °C.

Martensite is a supersaturated solid solution of carbon trapped in a body-centered tetragonal structure, and it is considered metastable. The term “martensite” was named after the German scientist Adolf Martens, who first used it to describe the hard microscopic phase found in quenched steels [26]. Lath martensite (LM), typically formed during the quenching process of C-Mn-Si high-strength steels, consists of parallel arrays or stacks of plate-like or lath-shaped crystals [27]. According to the ISIJ Bainite Committee classification, several microstructures are identified in low-carbon microalloyed steels, including two types of bainitic microstructures: granular ferrite and bainitic ferrite, often referred to as granular bainite and lath-like bainite (LB), respectively [28]. The LB defined by the ISIJ Bainite Committee classification consists of highly dislocated ferrite laths separated by low-angle boundaries, with M/A constituents interspersed between them. The microstructures of the test steels quenched at four different temperatures are shown in Figure 7. The test steel quenched at 950 °C primarily consisted of LM with a small amount LB. The amount of LB decreased while the content of LM increased as the *T*_wq_ increased to 1000 °C. When the *T*_wq_ was further raised to 1050 °C, the test steel consisted entirely of LM. For the steel quenched at 1100 °C, the prior austenite grains and lath bundles of LM became coarser, although the microstructure was still composed of martensite. As the holding temperature increases, the austenite grains grow larger and their stability improves. Consequently, the phase transformation during the cooling stage shifts toward lower temperatures, suppressing the bainite transformation and reducing the amount of LB.

**Figure 8:**
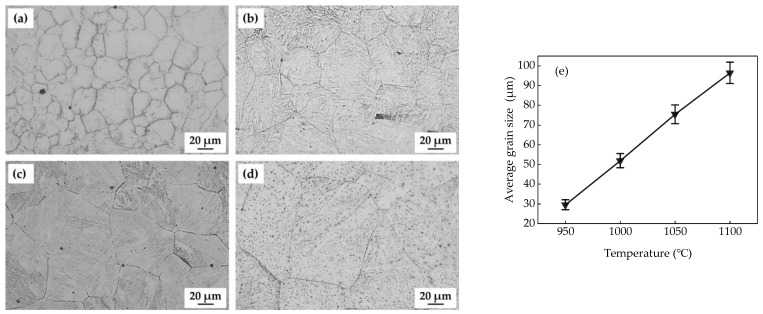
Grain structures and average grain size of the test steels at different *T*_wq_ levels: (**a**) 950 °C, (**b**) 1000 °C, (**c**) 1050 °C, (**d**) 1100 °C; (**e**) average grain size.

Figure 8 shows the grain size distribution in the test steels quenched at different temperatures. The average grain sizes of the test steels quenched at different temperatures were statistically analyzed, as shown in Figure 8e. The average grain sizes of the steels with different *T*_wq_ levels were 29.61 μm, 51.92 μm, 75.43 μm, and 96.52 μm, respectively. The average grain size of the test was observed to increase approximately linearly as the *T*_wq_ increased from 950 °C to 1100 °C. When the *T*_wq_ increased from 950 °C to 1000 °C, the average grain size increased from 29.61 μm to 51.92 μm with an increment of 75.34%. For 1100 °C-quenched steel, the average grain size was approximately 96.52 μm, which was about 3.26 times that of the 950 °C-quenched steel.

**Figure 9:**
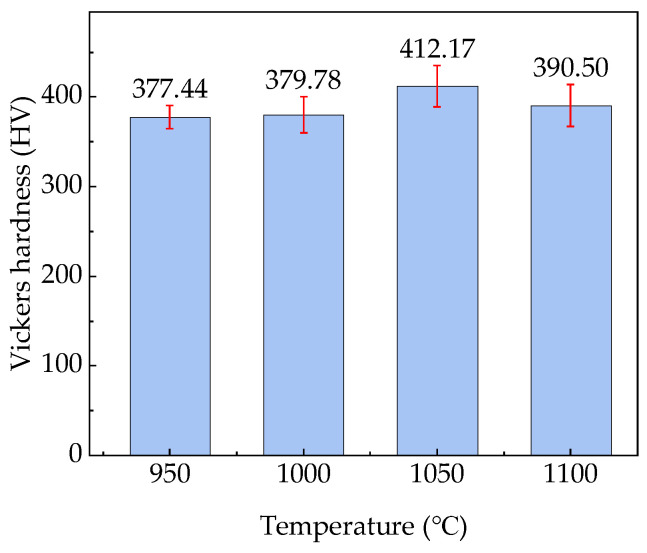
Hardnesses of the test steels at different *T*_wq_ levels.

Figure 9 shows the hardness test results of the test steels quenched at different temperatures. The hardness of the test steel increased with the *T*_wq_ and reached its maximum with a *T*_wq_ of 1050 °C, and then decreased slightly with the *T*_wq_. The hardnesses of the steels quenched at 950 °C and 1000 °C exhibited little variation with the values of 377.44 HV and 379.78 HV, respectively, with a 0.62% increase from 950 °C to 1000 °C. When *T*_wq_ increased to 1050 °C, the hardness increased to 412.17 HV with an increment of 34.73 HV compared to that of 950 °C-quenched steel, representing a 9.20% increase. However, the hardness decreased to 390.50 HV as *T*_wq_ increased to 1100 °C, a 5.26% decrease from the value at 1050 °C. At the *T*_wq_ levels of 950 °C and 1000 °C, LB existed in the test steel. The hardnesses of the test steel at these two temperatures were relatively low because the hardness of LB was lower than that of LM. As the *T*_wq_ increased to 1050 °C, the test steel was mainly composed of LM. Since the hardness of LM was relatively high, the hardness of the test steel reached a higher level. However, although the test steel was also mainly composed of LM when *T*_wq_ increased to 1100 °C, as shown in Figure 8, the average grain size of the test steel at 1100 °C was 96.52 μm, which was larger than 75.43 μm at 1050 °C. The 1050 °C-quenched steel exhibited a higher hardness than that of 1100 °C-quenched steel, owing to its higher dislocation density with smaller grains.

### 3.2. Pre-Charged Hydrogen Slow Strain Rate Tensile Test

**Figure 10:**
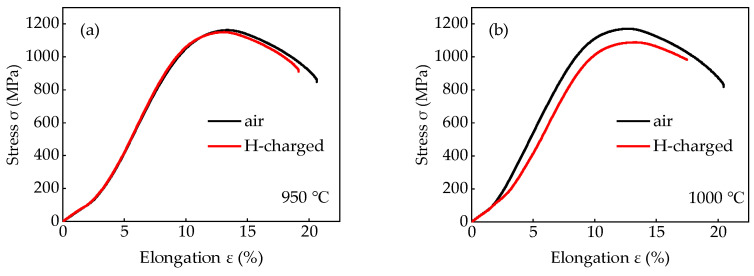

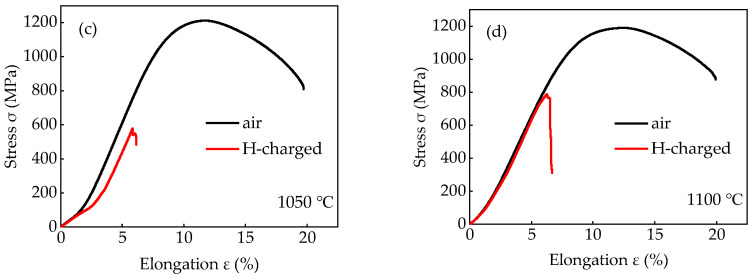
Stress–strain curves of the test steels at different *T*_wq_ levels: (**a**) 950 °C, (**b**) 1000 °C, (**c**) 1050 °C, (**d**) 1100 °C.

Figure 10 illustrates the stress–strain curves of the test steels with different *T*_wq_ levels. At the *T*_wq_ of 950 °C, hydrogen pre-charging induced a slight difference in the stress–strain curves between the hydrogen pre-charged tensile sample and the air-tensiled sample. At the *T*_wq_ levels of 1000 °C, 1050 °C, and 1100 °C, the reduction in tensile strengths with hydrogen pre-charging was more pronounced compared to that of the 950 °C-quenched tensiled samples. For the air-tensiled samples without hydrogen pre-charging, the tensile strengths of the test steels increased initially and then decreased, reaching values of 1163 MPa, 1171 MPa, 1213 MPa, and 1190 MPa, respectively. The peak value of 1213 MPa was reached at the *T*_wq_ of 1050 °C. However, with hydrogen pre-charging, the tensile strengths of the test steels decreased to 1150 MPa, 1089 MPa, 579 MPa, and 786 MPa, respectively. The tensile strengths of the steels were deteriorated to some extent with a minimum value of 579 MPa for *T*_wq_ of 1050 °C. With hydrogen pre-charging, the elongation rates were 19.1%, 17.5%, 6.1%, and 6.7%, respectively.

**Figure 11:**
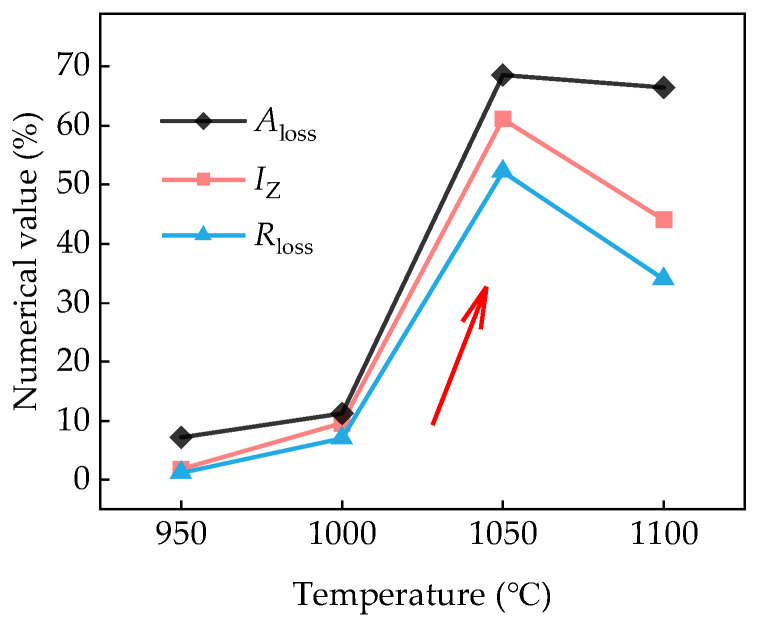
Loss in tensile properties and hydrogen embrittlement index *I*_z_ of the test steels with hydrogen pre-charging.

Figure 11 shows the tensile strength loss *R*_mloss_, the elongation loss *A*_loss_, and the *I*_z_ of the test steels after hydrogen charging. The red arrow indicates the direction towards the major probability of HIC susceptibility. At the *T*_wq_ of 950 °C, the *R*_mloss_ and *A*_loss_ of the test steel were 1.12% and 7.26%, respectively, with the *I*_z_ of 1.82%. As *T*_wq_ increased to 1000 °C, the *R*_mloss_ and *A*_loss_ of the test steel were 7.00% and 11.24%, respectively, with the *I*_z_ of 9.55%. For the *T*_wq_ of 1050 °C, the *R*_mloss_, *A*_loss_, and *I*_z_ of the test steel reached their maximum values of 52.27%, 68.62%, and 61.06%, respectively. When the *T*_wq_ increased to 1100 °C, the *R*_mloss_, *A*_loss_, and *I*_z_ of the test steel decreased slightly compared to that of 1050 °C, with values of 33.95%, 66.47%, and 55.02%, respectively. As the *T*_wq_ increased, the *R*_mloss_, *A*_loss_, and *I*_z_ of the test steels followed a similar varying regularity of first increasing and then decreasing. For the *T*_wq_ levels of 1050 °C and 1100 °C, the *I*_z_ values exceed the critical value of 35% defined by the evaluation standard [29], indicating a significant HIC susceptibility.

**Figure 12:**
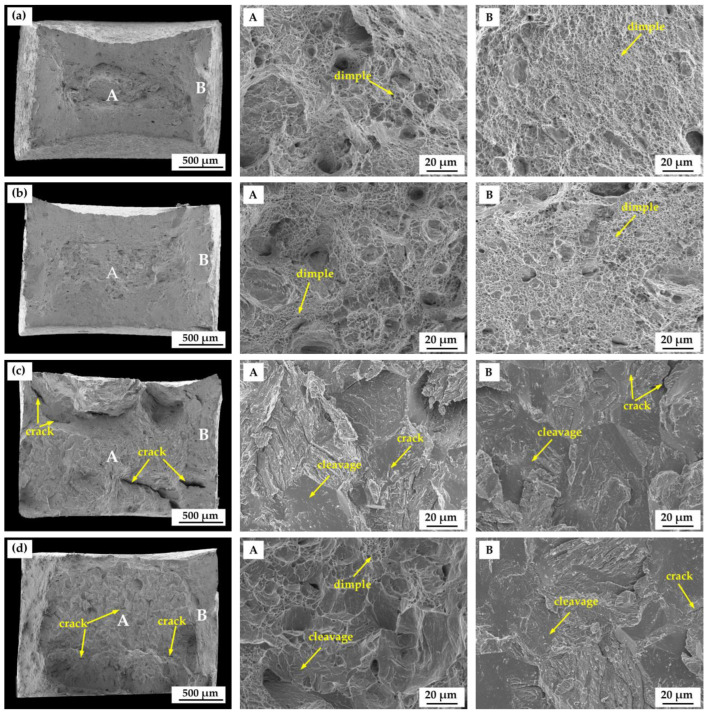
Fracture surface of the test steels after hydrogen charging at different *T*_wq_ levels: (**a**) 950 °C, (**b**) 1000 °C, (**c**) 1050 °C, (**d**) 1100 °C.

Figure 12a–d shows the fracture surfaces of water-quenched SSRT samples with hydrogen charging. As shown in Figure 12a,b, in the *T*_wq_ range of 950 °C to 1050 °C, the fracture morphology exhibited less necking as the *T*_wq_ increased. At the *T*_wq_ levels of 1050 °C and 1100 °C, the fracture morphologies exhibited typical brittle fracture morphology with almost no necking characteristics. Since hydrogen diffusion occurs at the surface layers of the alloy, fractography should also focus on the outer layers, so we carried out magnified observations of the B region, which is close to the fracture surface and can reflect the outer layers’ situation to some extent. High magnification fracture morphologies of central zone A and edge zone B were analyzed and are shown in Figure 12a,b. Dimples were identified both in the central and edge zones of the fracture surfaces for samples quenched at 950 °C and 1000 °C, while cleavage cracks were observed in the edge and the central zones of the fracture surfaces for samples quenched at 1050 °C and 1100 °C. At the *T*_wq_ of 1050 °C, the overall fracture surface exhibited prominent brittle fracture features of cleavage steps and river pattern, with approximately no visible dimples. As the *T*_wq_ was raised from 1050 °C to 1100 °C, shallow dimple features were redetected in the central zone of the fracture surface, with cleavage cracks still in the edge zone indicating a mainly brittle fracture. Compared to the samples quenched at 950 °C and 1000 °C, the number and size of the dimples in 1100 °C-quenched sample reduced, while the number of microcracks relatively increased.

### 3.3. Hydrogen Permeation

**Figure 13:**
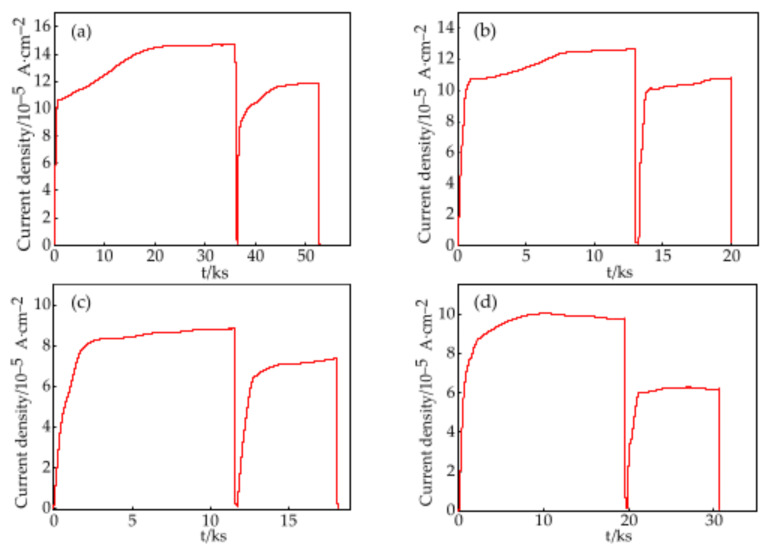
Hydrogen permeation curves of the test steels at different *T*_wq_ levels: (**a**) 950 °C, (**b**) 1000 °C, (**c**) 1050 °C, (**d**) 1100 °C.

The hydrogen permeation curves for the steels quenched at different temperatures are shown in Figure 13. The values of hydrogen permeation time and peak current densities of the first cycles were higher than those of the second cycles. The shorter permeation times of the second cycles were attributed to the hydrogen trapping only by reversible hydrogen traps [12]. Table 3 presents the calculated electrochemical hydrogen diffusion parameters for the steels quenched at different temperatures. As the *T*_wq_ increased, the *J*_∞_ and the *D*_eff_ initially decreased and then increased, reaching their minimum values at the *T*_wq_ of 1050 °C, with a *J*_∞_ of 8.76 × 10^−10^ mol·cm^−2^·s^−1^ and *D*_eff_ of 2.08 × 10^−6^ cm^2^·s^−1^. The calculated values of *C*_0_ of the test steels were 3.67 × 10^−5^ mol·cm^−3^, 3.71 × 10^−5^ mol·cm^−3^, 4.20 × 10^−5^ mol·cm^−3^, and 4.17 × 10^−5^ mol·cm^−3^, showing a maximum value occurring at the *T*_wq_ of 1050 °C. Generally, smaller values of *J*_∞_ and *D*_eff_ indicate a higher hydrogen trapping efficiency in the material [30]. It can be inferred that the steel quenched at 1050 °C with the smallest *J*_∞_ and *D*_eff_ exhibited the highest hydrogen capture efficiency. It is reported by Yazdipour [31] that grain sizes larger than 46 μm might induce lower hydrogen diffusivity owing to fewer diffusion routes with decreased grain boundary areas. For samples quenched at temperatures higher than 1000 °C, *D*_eff_ values were lower than that of the 950 °C-quenched sample. Fewer diffusion routes owing to their grain sizes greater than 50 μm might be one the factors inducing smaller *D*_eff_ values.

### 3.4. Hydrogen Microprint Technique and Hydrogen-Charged Cracking

**Figure 14:**
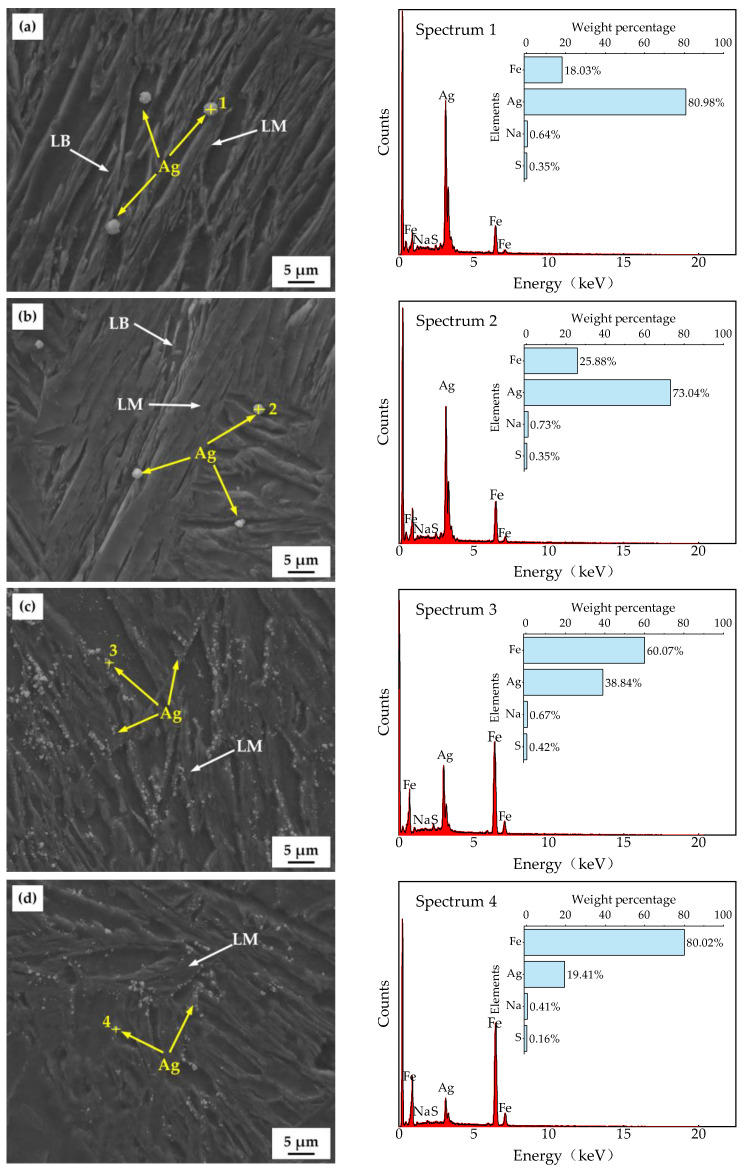
Hydrogen microprint technique images and EDS of the test steels at different *T*_wq_ levels: (**a**) 950 °C, (**b**) 1000 °C, (**c**) 1050 °C, (**d**) 1100 °C.

Figure 14 shows the corresponding EDS results of the water-quenched samples after the HMT tests. The test uses the reaction between hydrogen and AgBr (AgBr + H → Ag + HBr) to replace hydrogen in the metal and visualize hydrogen escape sites as silver particles. This reaction exhibits selectivity towards hydrogen escape sites. Furthermore, silver particles are easy to identify under microscopy due to their distinct contrast and morphology, which is ideal for visualization. The white particles can be confirmed as silver particles based on the EDS analysis results. The images revealed that white silver particles were mainly distributed along the martensitic lath interfaces, indicating a large number of hydrogen traps at these locations.

**Figure 15:**
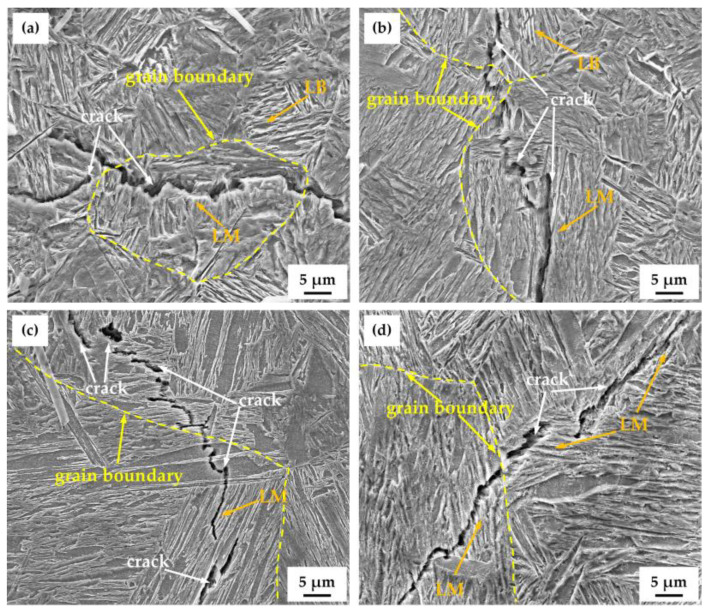
Images of cracks in test steels after hydrogen charging at different *T*_wq_ levels: (**a**) 950 °C, (**b**) 1000 °C, (**c**) 1050 °C, (**d**) 1100 °C.

Figure 15 shows the images of cracks in the four water-quenched samples after hydrogen charging. It is observed from Figure 15a,b that no cracks are observed in the bainitic structures. The cracks were located at the martensitic lath interfaces, indicating a greater HIC susceptibility of martensite than that of bainite. This is consistent with the findings of Jo [32]. It was reported that martensite lath interfaces had relatively high binding energies around 60 kJ/mol and could be classified as irreversible hydrogen traps [33]. Research has shown that austenite grain boundaries [34] and martensite lath structures [35] provide trapping sites for hydrogen atoms, which have a significant impact on HIC. It can also be seen from Figure 15 that the cracks in the four water-quenched samples were essentially transgranular cracks, and there was hardly any intergranular cracking identified. Thus, it can be concluded that martensitic lath interfaces were preferential sites for hydrogen trapping and crack propagation in the water-quenched samples studied in the present work.

## 4. Discussion

With the *T*_wq_ increasing from 950 °C to 1050 °C, the *I*_z_ of the quenched sample increased and reached a maximum value of 61.06% at 1050 °C. The 1050 °C-quenched sample with an intermediate average grain size (75.43 μm) exhibited the highest HIC susceptibility, hardness, *C*_0_, *N*_T_, *N*_r_, and *N*_ir_, and the lowest *D*_eff_. However, the *I*_z_ and hardness of the quenched sample decreased slightly with the *T*_wq_ increasing from 1050 °C to 1100 °C, although the grain size still increased with the *T*_wq_. This finding is consistent with that obtained by Sharma [36] in their research of the HIC behavior of X70 steel weldments, although Masoumi et al. [37] obtained contradictory results suggesting that finer-grained materials had a higher HIC susceptibility. It is concluded that grain size is not the single factor affecting the HIC susceptibility of the quenched steels with the *T*_wq_ varying in the range of 950 °C to 1050 °C. Microstructure is also a key factor influencing hydrogen trapping, diffusion, and HIC behavior [38]. The deterioration of HIC resistance with the *T*_wq_ increasing from 950 °C to 1050 °C can be attributed to microstructure variation with the *T*_wq_. At the *T*_wq_ levels of 950 °C and 1000 °C, the microstructures of the test steels were primarily composed of LB and LM. When the *T*_wq_ increased to 1050 °C and 1100 °C, the test steels were primarily composed of LM. Previous studies have shown that LM with high hardness contains substantial hydrogen traps and exhibits a higher HIC susceptibility compared to bainitic structures [31,39,40]. This is also confirmed by the findings of HMT and hydrogen-charged cracking tests conducted for the four groups of quenched samples. A higher volume percent of LM in 1050 °C-quenched steel than that in samples quenched at 950 °C and 1000 °C resulted in a higher HIC susceptibility and higher hardness. As for the *T*_wq_ above 1050 °C, grain growth is a decisive factor affecting the variation in HIC susceptibility and hardness. Research has confirmed the dependence of hardness and dislocation density on prior austenite grain size, and the dislocation density in smaller grains is higher than that of larger grains [41]. The 1050 °C-quenched steel exhibited a higher hardness, tensile strength and HIC susceptibility than that of 1100 °C-quenched steel, owing to its higher dislocation density with smaller grains. According to the internal hydrogen pressure (IHP) theory, when hydrogen atoms permeate metal materials, they tend to accumulate at grain boundaries, dislocations, or other defects, creating localized hydrogen pressure. This localized hydrogen pressure destabilizes the lattice, making it easier for cracks to initiate and propagate under the influence of hydrogen. The hydrogen pressure causes stress concentration in localized regions of the material, reducing its resistance to cracking and potentially leading to brittle fracture at lower stress levels.

In addition to the microstructure, hydrogen is easily captured by crystal defects (dislocations, vacancies, grain boundaries, etc.) during the diffusion process. These hydrogen traps have a significant impact on the hydrogen diffusion behavior [42]. Hydrogen traps with binding energies below 60 kJ/mol are classified as reversible hydrogen traps, including substitutional atoms, microvoids, dislocations, and grain boundaries. Among these, defects such as grain dislocations and boundaries can both act as trapping points for hydrogen and serve as channels for hydrogen diffusion [43]. The data in Table 3 indicate that *N*_r_ values were higher than *N*_ir_ for the quenched steels with different *T*_wq_ levels, suggesting that reversible hydrogen traps had a significant impact on hydrogen permeation. Research has found that martensitic laths possess a high density of grain boundaries, dislocations, residual stresses, and other defects. These microstructural features provide numerous reversible trapping sites for hydrogen adsorption and accumulation [44,45]. With the *T*_wq_ increasing from 950 °C to 1050 °C, increasing grain sizes resulted in decreasing grain boundaries as diffusion paths. The decreased *D*_eff_ value with the *T*_wq_ increasing from 950 °C to 1050 °C was therefore attributed to fewer diffusion channels and increasing hydrogen traps generated by LM. With the *T*_wq_ increasing from 1050 °C to 1100 °C, the increased *D*_eff_ value was mainly attributed to decreased hydrogen traps like grain boundaries, dislocations, and martensite lath interfaces.

## 5. Conclusions

This study examined the microstructure and HIC variation susceptibility in S355 steel at different *T*_wq_ levels using various experimental methods, including microstructural observation, pre-charged hydrogen SSRT test, HP test, and HMT test. The following conclusions are drawn:

(1) Based on the SSRT and HP tests, the test steel exhibited the highest HIC susceptibility at the *T*_wq_ of 1050 °C, manifesting a completely brittle fracture.

(2) According to the HMT and hydrogen-charged cracking tests, Ag predominantly accumulated along the martensitic laths. A significant number of hydrogen traps are present at the martensitic laths, leading to serious hydrogen accumulation in these areas.

(3) The HIC susceptibility in the quenched steels was influenced by various factors. At the *T*_wq_ of 950 °C and 1000 °C, the steels contained LB. The relatively lower HIC susceptibility of steels quenched at the two temperatures was mainly attributed to the HIC susceptibility of LB being lower than that of LM. At the *T*_wq_ levels of 1050 °C and 1100 °C, the test steels were basically composed of LM. The HIC susceptibility was higher in 1050 °C-quenched steel than that in the 1100 °C-quenched steel, accompanied by a greater number of hydrogen traps and elevated hardness.

## Figures and Tables

**Table 1:** Chemical composition of S355 steel (wt. %).

C	Si	Mn	P	S	Nb	Al	Cr	Cu	Ni	Fe
0.150	0.310	1.340	0.007	0.003	0.003	0.032	0.021	0.016	0.009	Balanced

**Table 2:** Mechanical properties of S355 steel.

Yield Strength/MPa	Tensile Strength/MPa	Elongation Rate/%	Impact Toughness at −20 °C/J
355~400	470~510	22~25	47~87

**Table 3:** Hydrogen permeation parameters of the test steels at different *T*_wq_ levels.

	Quenching Temperature/°C			
	950	1000	1050	1100
*I_∞_*/A cm^−2^	1.48 × 10^−9^	1.27 × 10^−9^	8.45 × 10^−10^	1.00 × 10^−9^
*J*_∞_/mol·cm^−2^·s^−1^	1.53 × 10^−9^	1.32 × 10^−9^	8.76 × 10^−10^	1.04 × 10^−9^
*D_eff_*/cm^2^·s^−1^	4.17 × 10^−6^	3.55 × 10^−6^	2.08 × 10^−6^	2.49 × 10^−6^
*C*_0_/mol·cm^−3^	3.67 × 10^−5^	3.71 × 10^−5^	4.20 × 10^−5^	4.17 × 10^−5^
*N_T_*/mol·cm^−3^	3.74 × 10^−3^	4.45 × 10^−3^	8.59 × 10^−3^	7.14 × 10^−3^
*N_r_*/mol·cm^−3^	2.04 × 10^−3^	2.72 × 10^−3^	5.74 × 10^−3^	3.89 × 10^−3^
*N_ir_*/mol·cm^−3^	1.70 × 10^−3^	1.73 × 10^−3^	3.25 × 10^−3^	2.85 × 10^−3^

## Data Availability

The original contributions presented in this study are included in the article. Further inquiries can be directed to the corresponding author.
